# Supplementary material for: Comparative Analysis of the Relative Fragmentation Stabilities of Polymorphic Alpha-Synuclein Amyloid Fibrils
Source: Biomolecules. 2022 Apr 25;12(5):630. doi: 10.3390/biom12050630 (PMC9138312; doi:10.3390/biom12050630)
Supplement: Supplementary file 1 [file biomolecules-12-00630-s001.zip › biomolecules-1639177-supplementary.pdf]

Comparative analysis of the relative fragmentation stabilities of polymorphic alpha-synuclein amyloid fibrils

SUPPLEMENTARY TABLE

Sarina Sanami[1], Tracey J. Purton[1], David P. Smith[2], Mick F. Tuite[1], Wei-Feng Xue[1*]

[1] Kent Fungal Group, School of Biosciences, Division of Natural Sciences, University of Kent, CT2 7NJ, Canterbury, UK; [2] Biomolecular Research Centre, Sheffield Hallam University, Sheffield, S1 1WB UK

* Correspondence to: W.F.Xue@kent.ac.uk

Key words: atomic force microscopy / amyloid / fibril fragmentation / fibril division / image analysis / sonication

Supplementary Table S1: Quantitative AFM image analysis statistics. Image analysis statistics for each fibril sample and sonication time point is shown.

	Fragmentation sonication time / s	*Number of images **	*Mean particle length / nm*	*Number of fibril particles †*	*Mean particle height / nm*	*Number of Pixels ‡*
WT	10	3	146.6	2356	7.8	124094
	20	2	159.7	3402	8.0	194749
	40	1	161.1	2122	7.8	121437
	80	3	91.0	11331	8.0	421682
	320	2	61.0	8462	7.6	217270
	640	2	56.9	8348	7.7	200526
A30P	10	3	232.3	1675	6.4	90746
	20	1	141.7	1333	6.1	63378
	40	2	91.3	3858	6.5	140956
	80	2	92.3	3354	6.4	119481
	320	1	68.3	2090	6.6	57942
	640	1	71.0	2069	6.6	60023
A53T	10	5	587.5	436	8.2	46284
	20	2	354.9	727	8.5	62580
	40	2	344.2	480	8.6	42181
	80	2	237.4	1447	7.2	94849
	320	3	196.8	2706	8.2	147446
	640	2	117.8	1911	8.6	75371

* All images are of 2048x2048 pixels covering 10 x 10 µm surface areas.

† Total number of fibril particles quantified for constructing the fibril length distributions.

‡ Total number of pixel height values measured for constructing the fibril height distributions.
